# A Duplex Proofman-LMTIA Assay for Rapid Detection of Shrimp, Crab, and Fish Allergens in Food Products

**DOI:** 10.3390/foods15213842

**Published:** 2026-10-27

**Authors:** Linqing Guo, Chaoqun Wang, Chunmei Song, Deguo Wang

**Affiliations:** 1 Key Laboratory of Biomarker Based Rapid-Detection Technology for Food Safety of Henan Province, Food and Pharmacy College, Xuchang University, Xuchang 461000, China; 13199571175@163.com (L.G.); 18203740159@139.com (C.W.); 2 Food College, Northeast Agricultural University, Harbin 150030, China; 3 Zhengzhou Customs District P. R. China, Technology Center, Zhengzhou 450000, China; 4 School of Public Health, Shandong Medical and Pharmaceutical University, Yantai 264003, China

**Keywords:** fish, shrimp, crab, allergen detection, multiple detection, ladder-shape melting temperature isothermal amplification, Proofman probe

## Abstract

Seafood allergy is a major global food safety concern, with fish, shrimp, and crab representing important allergenic sources. This study combined ladder-shape melting temperature isothermal amplification (LMTIA) with proofreading enzyme-mediated probe cleavage (Proofman) technology to establish a duplex assay for the rapid and simultaneous detection of crustacean (shrimp/crab) and fish allergens. Group-specific primers and Proofman probes were designed from conserved regions of shrimp and crab tropomyosin genes and the fish mitochondrial *16S rDNA* gene. The optimized reaction temperature was 61 °C, and amplification was completed within 30 min. The assay showed high specificity, with no cross-reactivity with squid, shellfish, or common livestock and poultry meats. The limits of detection for pure genomic DNA were 5 pg/μL for shrimp and fish and 10 pg/μL for crab. In model meat mixtures, the limits were 4% (*w*/*w*) for shrimp and 8% (*w*/*w*) for fish and crab. Results from 12 commercial processed foods were consistent with label declarations. Compared with the reference LAMP method specified in the Chinese Entry–Exit Inspection and Quarantine Industry Standard, the assay retained high sensitivity while enabling duplex detection and reducing nonspecific amplification, supporting its potential for rapid food safety testing.

## 1. Introduction

In the 21st century, food allergy has emerged as a major global public health concern. Although some children may outgrow their food allergies with age, most affected individuals experience persistent symptoms throughout their lives [1]. In recent years, the incidence of food allergy has shown an upward trend, with its global prevalence estimated at approximately 8% in children and 5% in adults [2,3]. A Chinese study reported an increase in the prevalence of food allergy among children, from 7.7% in 2009 to 11.1% in 2019 [4]. Following enactment of the Food Allergy Safety, Treatment, Education, and Research (FASTER) Act in the United States in 2021, sesame was officially added in 2023 as the ninth major food allergen, alongside milk, eggs, fish, crustacean shellfish, peanuts, soybeans, tree nuts, and wheat. These nine major allergens together account for approximately 90% of food allergy cases [5].

Shrimp, crab, and fish rank among the most widely consumed animal-protein sources worldwide. Their delicate texture, balanced amino acid profile, and high content of long-chain polyunsaturated fatty acids contribute to their importance in the human diet [6]. However, they are also major triggers of IgE-mediated allergic reactions. The clinical manifestations extend from mild skin irritation to life-threatening anaphylactic shock and severely affect the quality of life of allergic individuals [7]. Owing to the complexity of food-processing operations and raw-material supply chains, cross-contamination may occur at multiple stages, potentially resulting in the inadvertent consumption of allergens and consequent health risks. Therefore, developing rapid, sensitive, specific, and user-friendly detection methods is essential to safeguarding consumer health.

Considerable advances in food allergen detection over recent decades have resulted in methods broadly classified into protein-based and DNA-based approaches [8]. Among the protein-based methods currently in use are enzyme-linked immunosorbent assay (ELISA), lateral flow immunoassay (LFIA), and mass spectrometry (MS) [5,9]. Among immunological methods for food allergen detection and analysis, ELISA and LFIA are used most widely. The double-antibody sandwich ELISA relies on the combined action of a capture antibody and an enzyme-labeled detection antibody to generate a measurable optical density signal, thereby enabling qualitative and quantitative analysis of the target [10]. LFIA uses capillary action to transport a sample through different zones of a test strip, allowing separation, reaction, and signal readout to occur simultaneously as the sample migrates along the membrane. Although LFIA is simple and rapid and provides readily interpretable results, it is generally less sensitive than methods such as ELISA [11]. In highly processed foods, however, allergens are often exposed to treatments such as high temperatures, high pressures, and enzymatic hydrolysis, which may denature allergenic proteins and produce false-negative results in immunological assays. MS can effectively overcome the limitations of immunological methods but requires expensive instrumentation and specialized personnel, resulting in high detection costs [12].

The principal DNA-based detection methods are PCR, real-time PCR (qPCR), droplet digital PCR (ddPCR), and loop-mediated isothermal amplification (LAMP) [13,14]. These methods indirectly detect allergenic ingredients in foods by targeting group-specific gene sequences from the allergen source [15]. Conventional PCR offers advantages such as low cost and operational simplicity; however, it involves cumbersome procedures and requires opening of the tubes after amplification, which poses a risk of aerosol contamination [16]. qPCR enables one-step detection but still requires a thermal cycler, limiting its suitability for convenient on-site testing. ddPCR, a more recently developed technique, achieves absolute quantification by partitioning the reaction mixture into tens of thousands of individual droplets in which amplification occurs independently. However, the high cost of the required instrumentation precludes its use for on-site testing. LAMP enables isothermal amplification without thermal cycling, thereby facilitating on-site detection. Nevertheless, it requires four to six primers, complicating primer design, and is susceptible to primer-dimer formation and nonspecific amplification [17].

Ladder-shape melting temperature isothermal amplification (LMTIA) is based on differences between the melting temperatures of the primers and the target sequence. It uses a high-Tm folding primer and a low-Tm displacement primer to establish a temperature gradient, enabling isothermal amplification by Bst DNA polymerase [18]. The detailed amplification and signal generation mechanisms are illustrated in Figure S1. Relative to real-time PCR, LMTIA does not require a thermal cycler, reduces the reaction time to approximately 20 min, and offers high sensitivity; these characteristics make it suitable for rapid and convenient on-site detection. Compared with LAMP, LMTIA requires only two to four primers, can efficiently amplify target sequences as short as 60 bp, and exhibits exceptionally high specificity. To date, LMTIA has been successfully applied in various fields, including meat authentication [19,20], identification of edible oil components [21], differentiation of corn and sweet potato starches [22], identification of traditional Chinese medicinal materials [23,24], and detection of foodborne pathogenic bacteria [25]. Proofman (proofreading enzyme-mediated probe cleavage) technology combines the proofreading activity of an exonuclease with a fluorescent probe, enabling specific recognition during amplification and effectively preventing false-positive signals caused by primer dimers. This study aims to develop a rapid and sensitive detection method by integrating Proofman technology with LMTIA. To validate the sensitivity and specificity of this novel approach and promote its practical use in food safety and related fields, its performance will be compared with that of the LAMP method specified in the relevant Chinese EntryExit Inspection and Quarantine Industry Standard.

## 2. Materials and Methods

### 2.1. Materials

A total of 30 food samples—white shrimp (*Penaeus vannamei*), crayfish (*Procambarus clarkii*), lobster (*Panulirus* spp.), mantis shrimp (*Oratosquilla oratoria*), yellow catfish (*Pelteobagrus fulvidraco*), grass carp (*Ctenopharyngodon idella*), common carp (*Cyprinus carpio*), silver pomfret *(Pampus argenteus*), hairtail (*Trichiurus lepturus*), crucian carp (*Carassius auratus*), catfish (*Silurus asotus*), Atlantic salmon (*Salmo salar*), European perch (*Perca fluviatilis*), and large yellow croaker (*Larimichthys crocea*), Chinese mitten crab (*Eriocheir sinensis*), stone crab (*Charybdis* spp.), swimming crab (*Portunus trituberculatus*), flower crab (*Portunus pelagicus*), clam (*Ruditapes philippinarum*), scallop (*Chlamys farreri*), oyster (*Crassostrea gigas*), razor clam (*Sinonovacula constricta*), squid (*Loligo* spp.), mussels (*Mytilus* spp.), abalones (*Haliotis* spp.), beef, mutton, chicken, duck, and goose—were purchased from the Pangdonglai Supermarket in Xuchang, China. Twelve processed products—shrimp balls, shrimp paste, dried shrimp, shrimp sauce, fish floss, fish balls, fish tofu, grilled fish fillets, cod sausage, shredded crab, crab paste, and crab sauce—were purchased from online supermarkets. The Animal Genomic DNA Extraction Kit (DP304-03) was obtained from Tiangen Biotech (Beijing) Co., Ltd., Beijing, China. Bst DNA polymerase was purchased from Kelaoning (Beijing) Biotechnology Co., Ltd., Beijing, China. Diethyl pyrocarbonate-treated water (DEPC-H_2_O) was purchased from Hefei Lair Biotechnology Co., Ltd., Hefei, China. The remaining reagents were purchased from Sinopharm Chemical Reagent Co., Ltd., Shanghai, China.

### 2.2. Design of Primers and Probes

**Figure 1:**
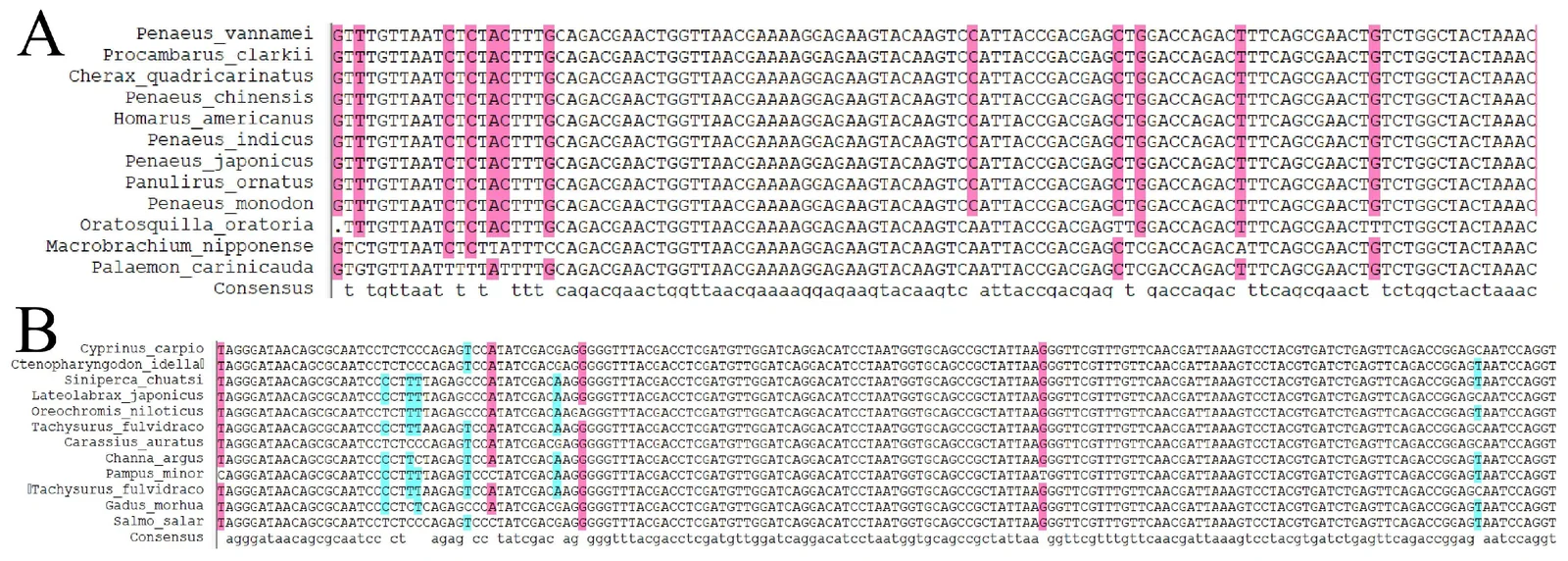
Multiple sequence alignments of the target regions used for primer and probe design. (**A**) Conserved regions of crustacean tropomyosin gene sequences. (**B**) Conserved regions of fish mitochondrial *16S rDNA* sequences. The colored highlights indicate nucleotide positions marked by the sequence alignment software to facilitate the visualization of sequence similarities and differences.

Conserved regions in the tropomyosin genes of shrimp and crab and in the fish mitochondrial *16S rDNA* sequences formed the basis for primer and probe design. The corresponding gene sequences from different shrimp, crab, and fish species were retrieved from the NCBI GenBank database (https://www.ncbi.nlm.nih.gov/genbank/, accessed on 5 February 2026), and the accession numbers are listed in Table 1. Multiple sequence alignments were performed using DNAMAN software (version 9.0.1.116; Lynnon Biosoft, San Ramon, CA, USA) to identify conserved regions (Figure 1A,B).

**Figure 2:**
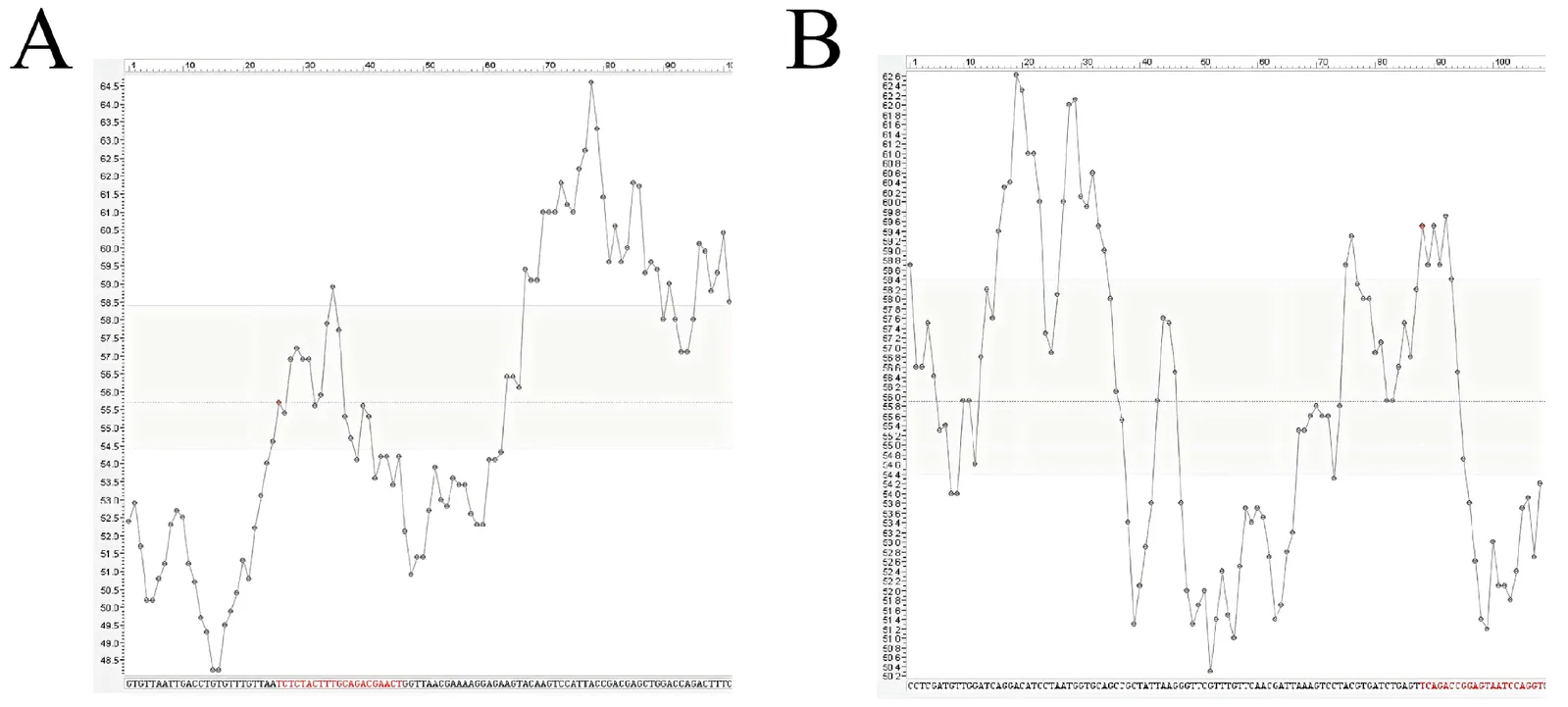
Melting-temperature profiles of the conserved target regions. (**A**) Melting-temperature curve of the conserved crustacean tropomyosin sequence. (**B**) Melting-temperature curve of the conserved fish mitochondrial *16S rDNA* sequence.

Candidate target regions exhibiting ladder-shape melting-temperature profiles were screened using Oligo 7 software (version 7.56; Molecular Biology Insights, Inc., Colorado Springs, CO, USA) (Figure 2A,B). LMTIA primers specific for the shrimp/crab and fish targets were designed using the Primer 3 Plus online tool (http://www.primer3plus.com, accessed on 21 February 2026). Two Proofman probes with different fluorescent labels were designed based on the corresponding loop-primer (LF/LB) sequences to enable duplex fluorescence detection. The shrimp/crab-specific probe was labeled with Cy5, whereas the fish-specific probe was labeled with FAM.

All primers and probes used in this study were synthesized by Anhui General Biotechnology Co., Ltd. (Chuzhou, China). Detailed primer and probe sequences are provided in Table 2.

The LAMP primers used for the reference shrimp detection assay were obtained from the relevant Chinese export-food allergen testing standard [26]. Primer sequences are shown in Table 3.

### 2.3. DNA Extraction

For each animal tissue sample, approximately 30 mg was collected and subjected to lysis in 200 μL of lysis buffer at 56 °C until the tissue was completely digested. Genomic DNA extraction was then performed following the manufacturer’s instructions for the Tiangen Animal Genomic DNA Extraction Kit.

A NanoDrop One spectrophotometer (Thermo Fisher Scientific, Waltham, MA, USA) was used to assess DNA purity. DNA samples were considered suitable for subsequent experiments when their A_260_/A_280_ ratios were between 1.8 and 2.0. A Qubit 4 fluorometer (Thermo Fisher Scientific, USA) was used to determine DNA concentrations. DNA samples intended for long-term storage were maintained at −80 °C, whereas samples used within a short period were stored at −20 °C.

### 2.4. Proofman-LMTIA Reaction System

The duplex Proofman-LMTIA reaction mixture contained LMTIA premix buffer (1×), crustacean (shrimp/crab)-specific primers (F1/B1/LF1/LB1), fish-specific primers (F2/B2/LF2/LB2), Bst DNA polymerase, the corresponding Proofman probes, DNA template, and DEPC-H_2_O. Detailed reagent concentrations and volumes are provided in Table 4.

For shrimp allergen detection, the LAMP reaction system was established in accordance with the Chinese Entry–Exit Inspection and Quarantine Industry Standard. The composition of the LAMP reaction system is presented in Table 5.

To minimize the risk of aerosol contamination, 20 μL of mineral oil was layered over each reaction mixture. A Gentier 96E real-time PCR system (Tianlong Technology, Xi’an, China) was used for amplification and real-time fluorescence acquisition.

### 2.5. Optimization of the Reaction Temperature for the Duplex Proofman-LMTIA Assay

The duplex reaction system described in Table 4 was used for temperature optimization. An equal-volume mixture of shrimp and fish genomic DNA was used as the positive control, and DEPC-H_2_O was used as the negative control. A Gentier 96E real-time PCR system (Tianlong Technology, Xi’an, China) was used to perform duplex isothermal amplification.

Based on preliminary optimization experiments with singleplex assays (Figure S2), the optimal reaction temperatures for shrimp and fish were determined to be 60 °C and 61 °C, respectively. Therefore, a temperature range of 59–62 °C, encompassing the optimal temperatures of both targets, was selected for further optimization of the duplex assay. Fluorescence signals were recorded every 45 s for a total of 40 acquisition cycles. Duplicate reactions were performed for each condition, and the experiment was repeated independently three times. Amplification efficiency, fluorescence intensity, and the consistency of the amplification curves were the criteria used to select the optimal reaction temperature.

### 2.6. Analytical Specificity of the Duplex Proofman-LMTIA Assay

The analytical specificity of the duplex Proofman-LMTIA assay was evaluated using genomic DNA extracted from a panel of target and non-target species. Genomic DNA from white shrimp (*Penaeus vannamei*), crayfish (*Procambarus clarkii*), lobster (*Panulirus* spp.), mantis shrimp (*Oratosquilla oratoria*), yellow catfish (*Pelteobagrus fulvidraco*), grass carp (*Ctenopharyngodon idella*), common carp (*Cyprinus carpio*), silver pomfret (*Pampus argenteus*), hairtail (*Trichiurus lepturus*), crucian carp (*Carassius auratus*), catfish (*Silurus asotus*), Atlantic salmon (*Salmo salar*), European perch (*Perca fluviatilis*), and large yellow croaker (*Larimichthys crocea*), Chinese mitten crab (*Eriocheir sinensis*), stone crab (*Charybdis* spp.), swimming crab (*Portunus trituberculatus*), and flower crab (*Portunus pelagicus*) was used as positive control.

Genomic DNA from clam (*Ruditapes philippinarum*), scallop (*Chlamys farreri*), oyster (*Crassostrea gigas*), mussels (*Mytilus* spp.), abalones (*Haliotis* spp.), razor clam (*Sinonovacula constricta*), squid (*Loligo* spp.), beef, mutton, chicken, duck, and goose was used as non-target DNA, and DEPC-H_2_O served as the no-template control.

Amplification was performed using the duplex reaction system described in Table 4 at the optimized reaction temperature. Fluorescence signals were recorded every 45 s for a total of 40 acquisition cycles. Each sample was tested in duplicate, and the experiment was independently repeated three times.

### 2.7. Repeatability of the Duplex Proofman-LMTIA Assay

For evaluation of both stability and repeatability of the duplex Proofman-LMTIA assay, DEPC-H_2_O served as the negative control, whereas shrimp, crab, and fish genomic DNA served as the positive control. Amplification was performed at the optimized reaction temperature, and fluorescence signals were recorded every 45 s for a total of 40 acquisitions. Ten parallel reactions were performed for each experimental condition.

The primary parameter used for qualitative evaluation of amplification performance was the cycle threshold (Ct) value. The relative standard deviation (RSD) of the Ct values was calculated to assess the stability and repeatability of the assay, with a lower RSD indicating better consistency. The RSD was calculated using the following equations:

(1) $${\mathrm{RSD}}_{Ct}=\frac{{\mathrm{SD}}_{Ct}}{{Mean}_{Ct}}\times 100\%$$

(2) $${SD}_{Ct}=\sqrt[{}]{\frac{\sum_{i=1}^{n}{\left({Ct}_{i}-\bar{Ct}\right)}^{2}}{n-1}}$$

### 2.8. Analytical Sensitivity of the Duplex Proofman-LMTIA Assay

To evaluate the analytical sensitivity of the duplex Proofman-LMTIA assay, shrimp, crab, and fish genomic DNA were separately serially diluted to concentrations of 1 ng/μL, 500 pg/μL, 100 pg/μL, 50 pg/μL, 10 pg/μL, 5 pg/μL, and 1 pg/μL. DNA at each concentration was used as a positive control, whereas DEPC-H_2_O served as the negative control.

Amplification was performed using the duplex reaction system described in Table 4 at the optimized reaction temperature. Fluorescence signals were recorded every 45 s for a total of 40 acquisition cycles. Each concentration was tested in duplicate, and the experiment was independently repeated three times.

### 2.9. Detection Limit of the Duplex Proofman-LMTIA Assay in Model Meat Mixtures

To assess the detection performance of the duplex Proofman-LMTIA assay in food matrices, shrimp, crab, and fish meats were each thoroughly mixed with chicken meat to prepare target mass fractions of 1%, 4%, 8%, 10%, 25%, 50%, 75%, and 95% (*w*/*w*). Three types of model meat mixtures were prepared: shrimp-chicken mixtures (Xia), crab-chicken mixtures (Xie), and fish-chicken mixtures (YuR). Genomic DNA was extracted from each model meat mixture according to the procedure described in Section 2.3. DNA extracted from the model mixtures was used as the test template, and DEPC-H_2_O was used as the negative control.

Amplification used the optimized duplex reaction system and reaction temperature, with fluorescence signals recorded every 45 s for a total of 40 acquisition cycles. Each sample was tested in duplicate, and the experiment was independently repeated three times. The lowest target mass fraction that consistently generated a positive amplification signal under the tested conditions was considered the minimum detectable mass fraction.

### 2.10. Analysis of Commercial Food Products

A total of 12 commercially processed products labeled as containing shrimp, crab, or fish ingredients were analyzed to evaluate the applicability of the duplex Proofman-LMTIA assay: fish floss, fish balls, fish tofu, grilled fish fillets, cod sausage, shredded crab products, crab paste, crab sauce products, shrimp balls, shrimp paste, dried shrimp, and shrimp sauce.

For each sample, approximately 30 mg was weighed, and genomic DNA was extracted following the manufacturer’s instructions for a DNA extraction kit for highly processed foods (DP326, Tiangen Biotech [Beijing] Co., Ltd., Beijing, China).

The DNA obtained from the commercial food samples served as the test template; shrimp/crab and fish genomic DNA served as the positive control, and DEPC-H_2_O served as the negative control. The optimized duplex reaction system and reaction temperature were used for amplification. Fluorescence signals were recorded every 45 s for a total of 40 acquisition cycles. Duplicate analyses were conducted for each sample, with three independent repetitions of the experiment.

### 2.11. Reference LAMP Assay for Shrimp Detection

For shrimp detection, the LAMP assay followed the Chinese Export Food Allergen Testing Standard [26] and used the reaction system specified in Table 5.

For analytical sensitivity testing, shrimp genomic DNA diluted to 1 ng/μL, 500 pg/μL, 100 pg/μL, 50 pg/μL, 10 pg/μL, 5 pg/μL, and 1 pg/μL served as the positive controls. DEPC-H_2_O was used as the negative control. LAMP was performed at the optimized reaction temperature of 63 °C for 1 h. Each DNA concentration was tested in duplicate, and the experiment was independently repeated three times.

## 3. Results

### 3.1. Temperature Optimization of the Duplex Proofman-LMTIA Assay

**Figure 3:**
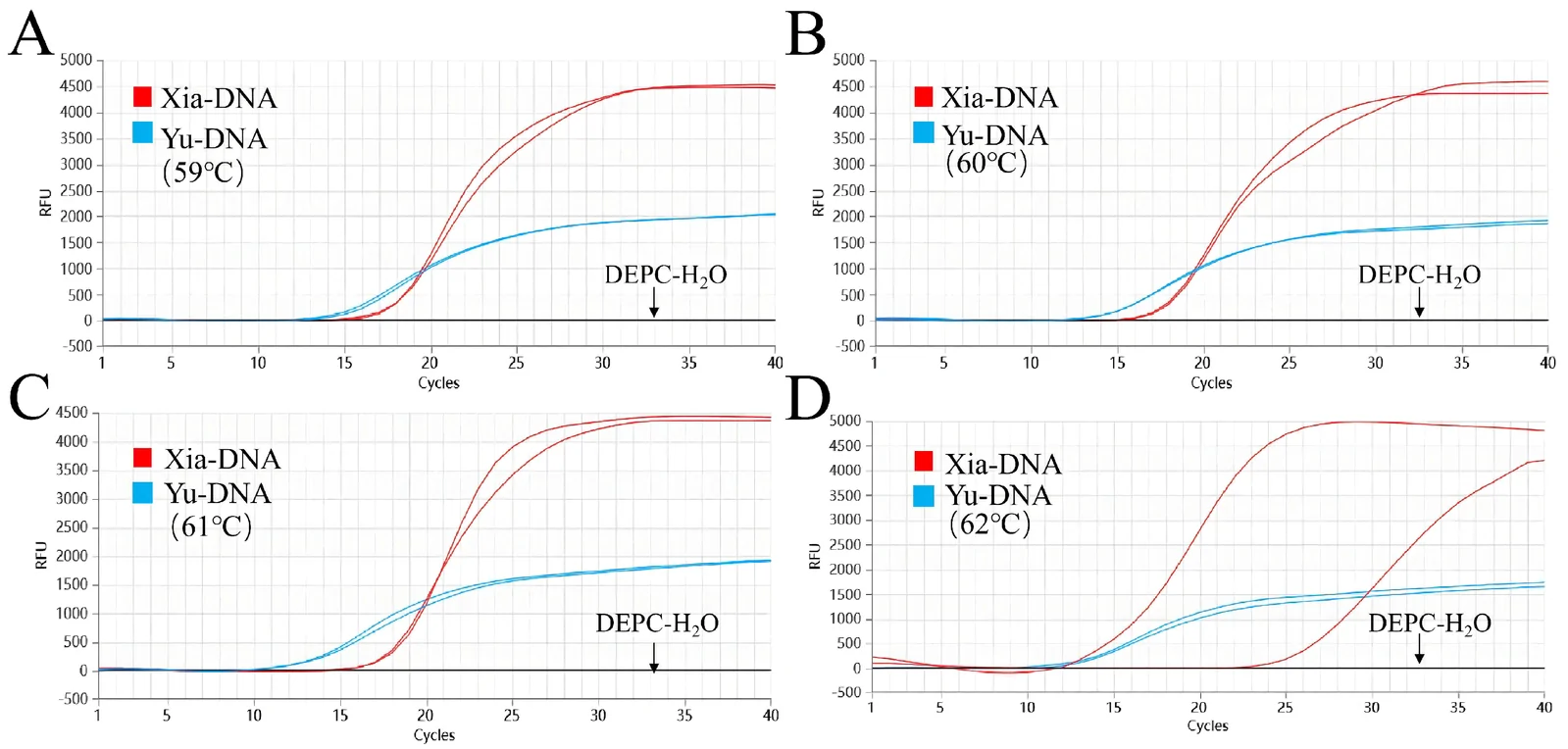
Reaction-temperature optimization for shrimp and fish using duplex Proofman-LMTIA. (**A**–**D**): Amplification curves at 59 °C, 60 °C, 61 °C, and 62 °C, respectively. Positive controls, shrimp (Xia) and fish (Yu) gDNA; negative control, DEPC-H_2_O. Xia: shrimp. Yu: fish.

The results of reaction-temperature optimization for the duplex Proofman-LMTIA assay are shown in Figure 3A–D. Across the tested temperatures of 59–62 °C, none of the negative controls showed amplification, whereas every positive control yielded a target-specific amplification signal. However, differences in amplification performance were observed among the four temperatures.

Based on a comprehensive comparison of cycle threshold (Ct) values, fluorescence intensity, and consistency of the amplification curves, the best overall amplification performance was obtained at 61 °C. At this temperature, fish genomic DNA generated a readily detectable amplification signal by approximately the 10th fluorescence-acquisition cycle. Accordingly, 61 °C was chosen as the optimal reaction temperature for the subsequent experiments.

### 3.2. Specificity Evaluation of the Duplex Proofman-LMTIA Assay

**Figure 4:**
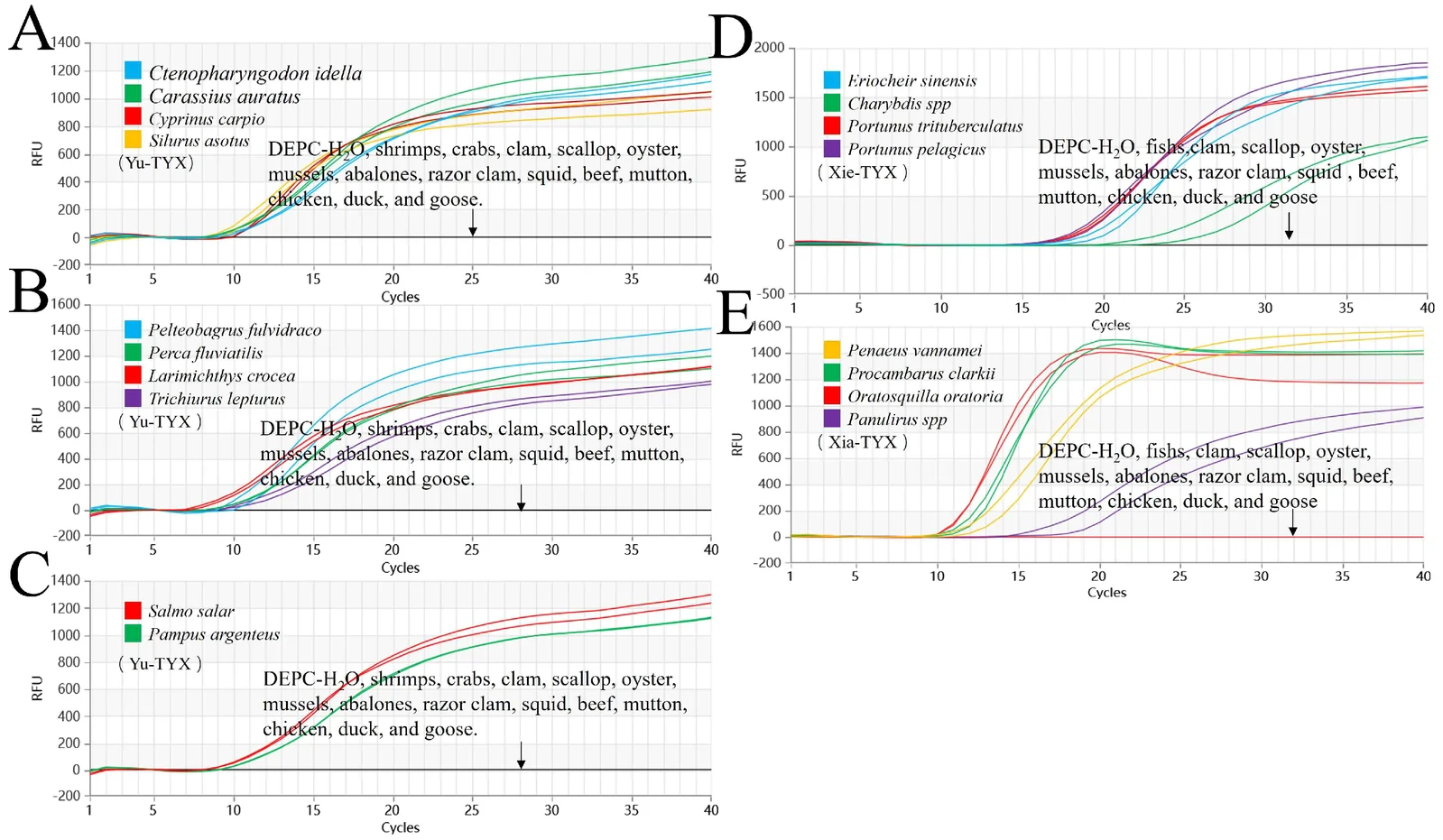
Assessment of duplex Proofman-LMTIA assay specificity for crustaceans and fish at 61 °C. (**A**–**C**): Fish-specific amplification curves for ten fish species. (**D**): Crustacean-specific amplification curves for four crab species. (**E**): Crustacean-specific amplification curves for four shrimp species. Negative controls, DEPC-H_2_O and the non-target samples indicated in each panel. Xia: shrimp. Xie: crab. Yu: fish.

Figure 4A–E present the analytical specificity findings for the duplex Proofman-LMTIA assay. No amplification signals were detected from genomic DNA of the non-target samples, including clam, scallop, oyster, mussels, abalones, razor clam, squid, beef, mutton, chicken, duck, and goose. No amplification was observed in the DEPC-H_2_O negative control.

In contrast, clear amplification signals were obtained from all tested shrimp (four species), crab (four species), and fish (ten species) samples. Shrimp and crab DNA generated amplification signals exclusively in the Cy5 channel, which was specific for the crustacean target, whereas fish DNA generated signals exclusively in the fish-specific FAM channel. No detectable cross-reactivity was observed between the two fluorescence channels. These results demonstrate that the established duplex Proofman-LMTIA assay showed good analytical specificity for the crustacean and fish species examined in this study.

### 3.3. Repeatability Assessment of the Duplex Proofman-LMTIA Assay

**Figure 5:**
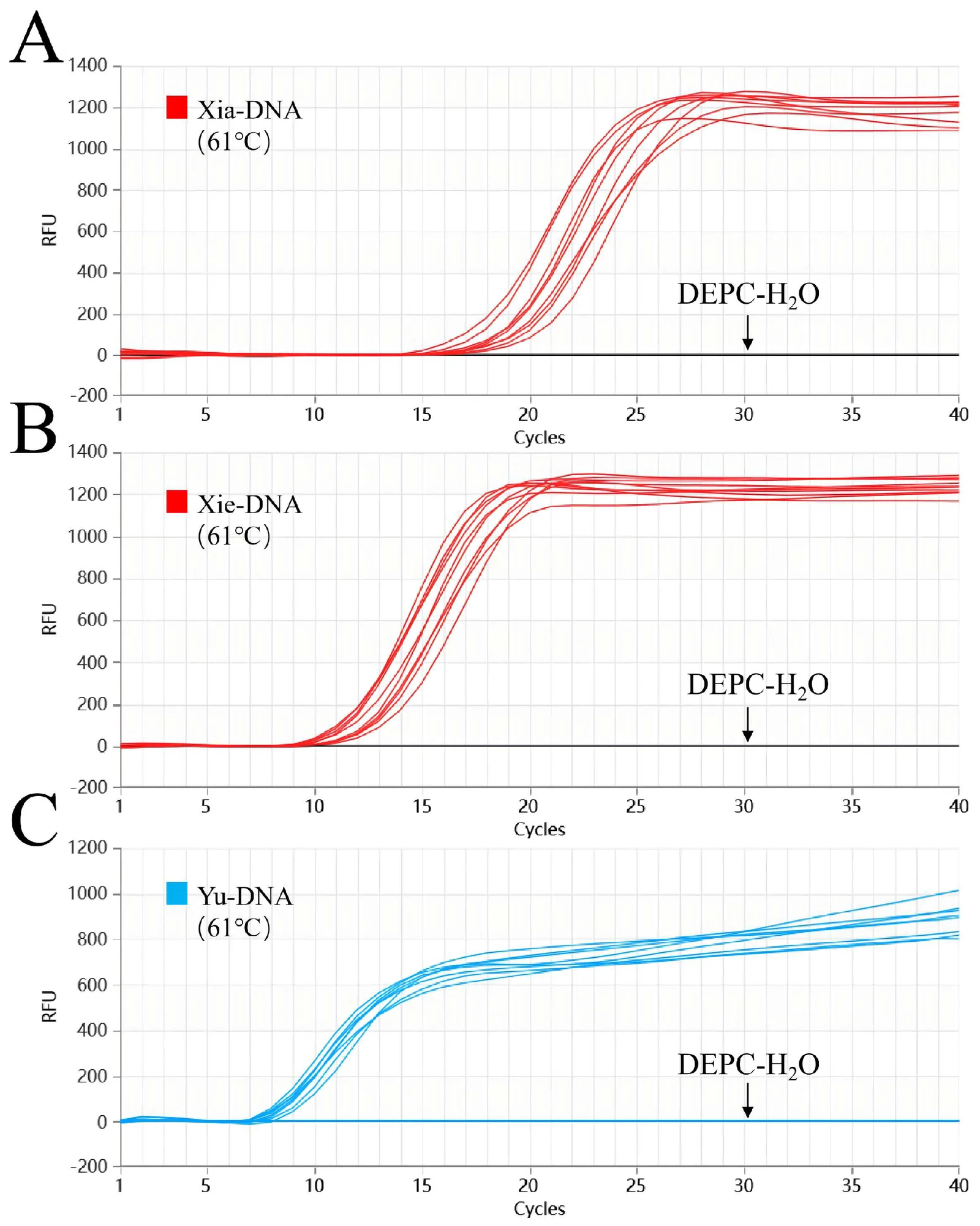
Repeatability assessment of the duplex Proofman-LMTIA assay at 61 °C for shrimp, crab, and fish. (**A**–**C**): Amplification curves from ten parallel reactions using shrimp, crab, and fish gDNA, respectively. Positive controls, shrimp, crab, or fish gDNA; negative control, DEPC-H_2_O. Xia: shrimp. Xie: crab. Yu: fish.

For repeatability assessment of the duplex Proofman-LMTIA assay, crustacean (shrimp/crab) and fish genomic DNA served as positive controls, with DEPC-H_2_O as the negative control. As shown in Figure 5A–C, clear amplification was obtained for all three target groups at the optimized reaction temperature of 61 °C, whereas no amplification was observed in the negative controls.

For ten parallel reactions, the cycle threshold (Ct) values (one acquisition cycle corresponds to 45 s) ranged from 17.2 to 20.5 cycles for shrimp, 11.5 to 14.3 cycles for crab, and 7.9 to 9.3 cycles for fish. The corresponding relative standard deviations (RSDs) were 6.1%, 5.2%, and 3.8%, respectively. The relatively low RSD values indicated limited variation among parallel reactions, demonstrating good repeatability of the duplex Proofman-LMTIA assay.

### 3.4. Sensitivity Evaluation of the Duplex Proofman-LMTIA Assay

**Figure 6:**
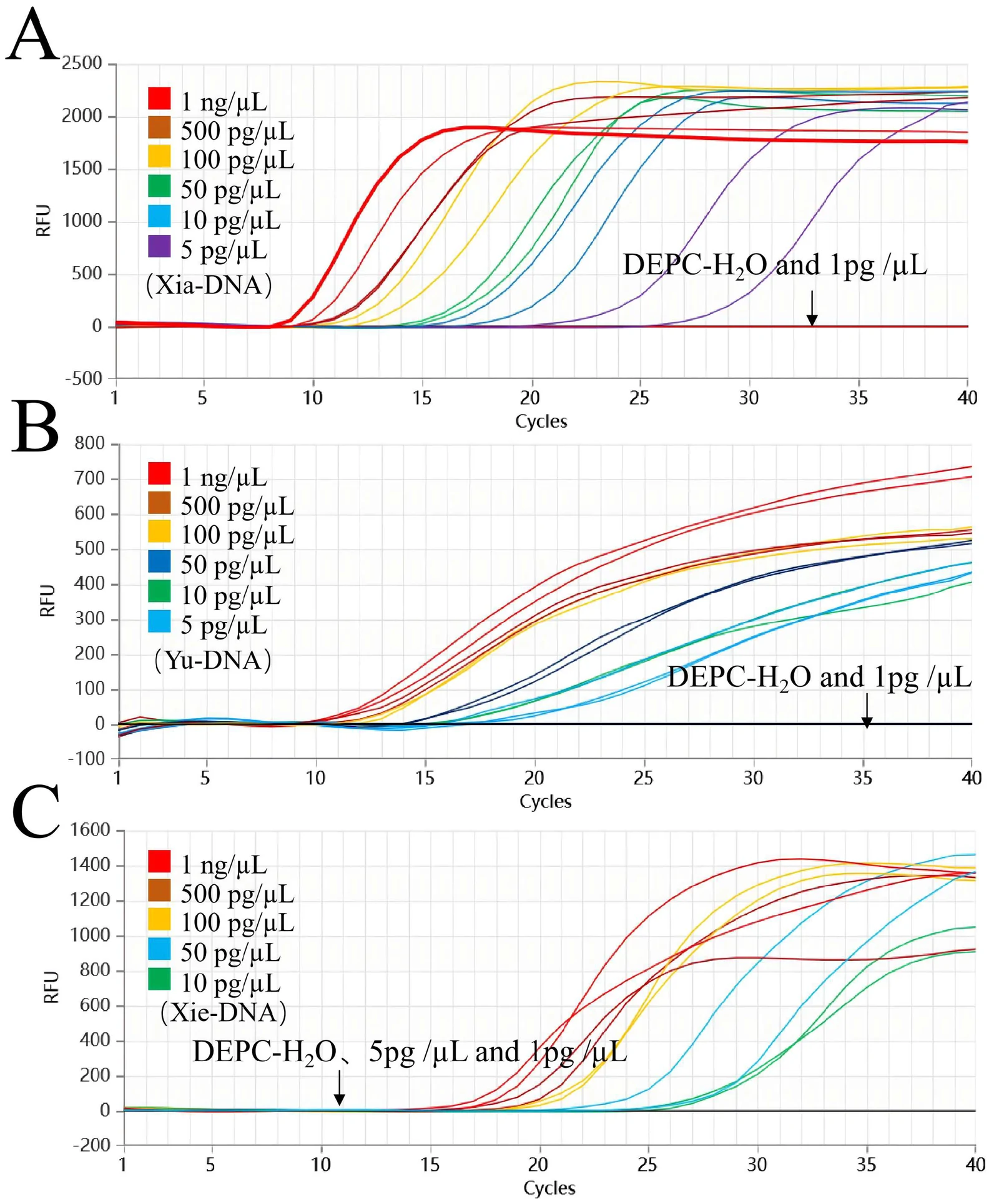
Analytical sensitivity of the duplex Proofman-LMTIA assay at 61 °C for shrimp, fish, and crab. (**A**–**C**): Amplification curves for shrimp, fish, and crab gDNA, respectively. The gDNA was tested at 1 ng/µL, 500 pg/µL, 100 pg/µL, 50 pg/µL, 10 pg/µL, 5 pg/µL, and 1 pg/µL. Negative control, DEPC-H_2_O. Xia: shrimp. Xie: crab. Yu: fish.

Figure 6A–C presents the analytical sensitivity results for the duplex Proofman-LMTIA assay. When tested at the optimized reaction temperature of 61 °C, shrimp genomic DNA generated positive amplification signals at concentrations of 1 ng/μL, 500 pg/μL, 100 pg/μL, 50 pg/μL, 10 pg/μL, and 5 pg/μL, whereas no amplification was detected at 1 pg/μL or in the negative control. The same detection pattern was observed for fish genomic DNA. For crab genomic DNA, positive amplification was observed at concentrations of 1 ng/μL, 500 pg/μL, 100 pg/μL, 50 pg/μL, and 10 pg/μL, whereas no amplification was detected at 5 pg/μL, 1 pg/μL, or in the negative control.

As the genomic DNA concentration decreased, the Ct values increased progressively and the fluorescence intensity tended to decrease. Under the predefined criterion of Ct < 35 for a positive reaction, the minimum detectable genomic DNA concentrations were 5 pg/μL for shrimp and fish and 10 pg/μL for crab.

### 3.5. Limit of Detection of the Duplex Proofman-LMTIA Assay

**Figure 7:**
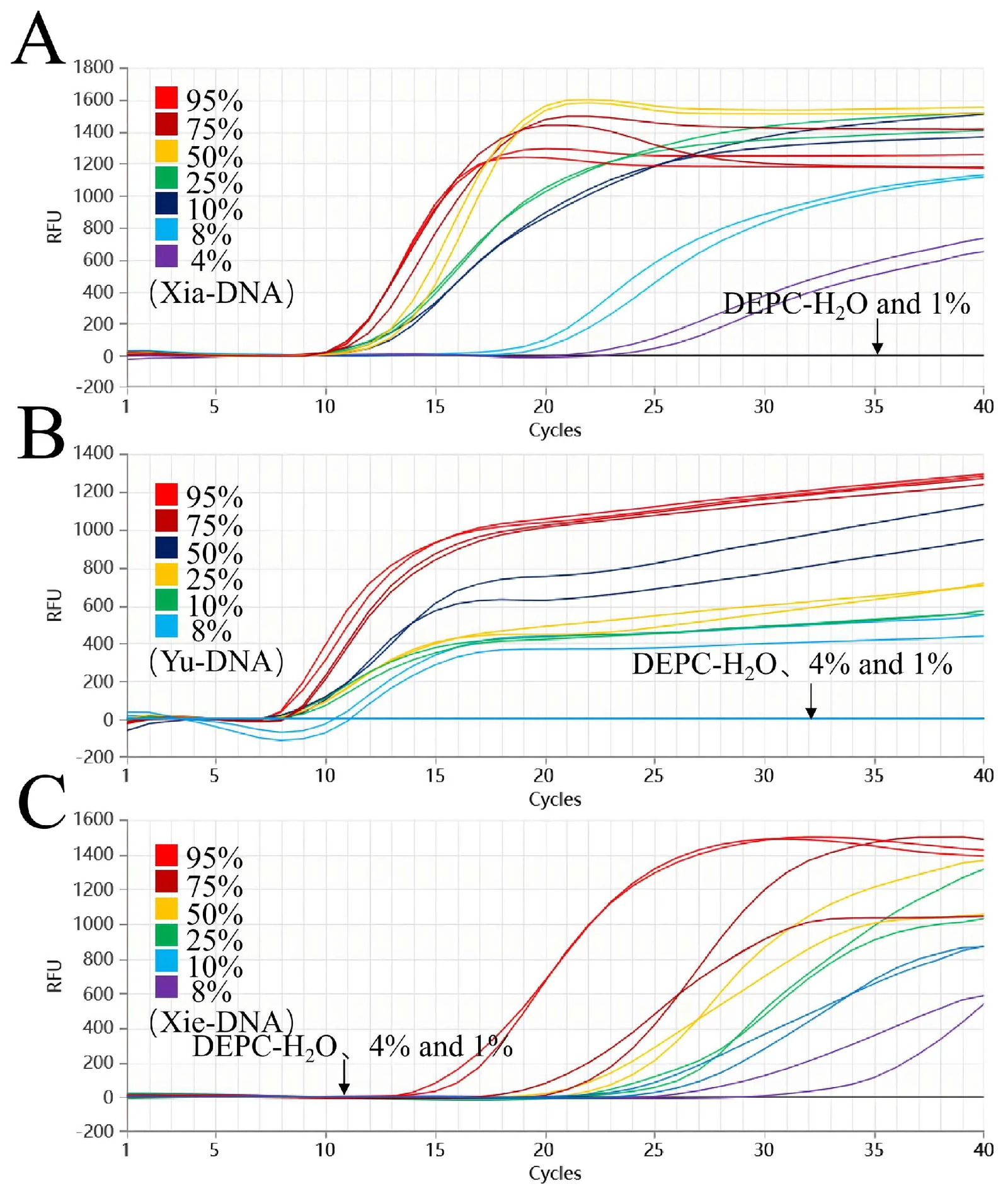
Detection-limit assessment of the duplex Proofman-LMTIA assay in model meat mixtures. (**A**–**C**): Amplification curves for shrimp-chicken, fish-chicken, and crab-chicken mixtures, respectively. The target mass fractions were 1%, 4%, 8%, 10%, 25%, 50%, 75%, and 95% (*w*/*w*). Negative control, DEPC-H_2_O. Xia: shrimp. Xie: crab. Yu: fish.

Figure 7A–C illustrates the detection limits of the duplex Proofman-LMTIA assay in model meat mixtures. For shrimp-containing mixtures, positive amplification signals were obtained at shrimp mass fractions of 4%, 8%, 10%, 25%, 50%, 75%, and 95% (*w*/*w*), whereas no amplification was detected at mass fractions of 1% or in the DEPC-H_2_O negative control. The minimum detectable shrimp mass fraction under the tested conditions was therefore 4% (*w*/*w*). For fish and crab-containing mixtures, positive amplification was observed at target mass fractions of 8% (*w*/*w*) and above, whereas samples containing 4% or 1% (*w*/*w*) target material and the corresponding negative controls did not exhibit amplification. Accordingly, the minimum detectable mass fraction was 8% (*w*/*w*) for both fish and crab.

Taken together, these findings indicate minimum detectable mass fractions of 4% (*w*/*w*) for shrimp and 8% (*w*/*w*) for fish and crab with the duplex Proofman-LMTIA assay. In addition, decreasing target mass fractions were generally associated with delayed threshold crossing, as reflected by progressively increased Ct values.

### 3.6. Detection of Allergens in Commercial Food Products

A total of 12 commercially available processed food products labeled as containing shrimp, crab, or fish ingredients were analyzed to assess the practical applicability of the duplex Proofman-LMTIA assay. The results are shown in Figure S3. All negative controls showed no amplification. The shrimp balls, shrimp paste, dried shrimp, and shrimp sauce produced positive signals in the crustacean (shrimp/crab)-specific Cy5 channel. Fish floss, fish balls, fish tofu, grilled fish fillets, and cod sausage produced positive signals in the fish-specific FAM channel. The shredded crab product, crab paste, and crab sauce also produced positive signals in the crustacean-specific Cy5 channel. The results obtained for all 12 commercial products were concordant with the allergenic ingredients declared on their product labels. These findings provide preliminary evidence supporting the application of the duplex Proofman-LMTIA assay for detecting crustacean (shrimp/crab) and fish-derived components in processed food matrices.

### 3.7. Sensitivity Evaluation of the LAMP Method for Shrimp Allergen Detection

**Figure 8:**
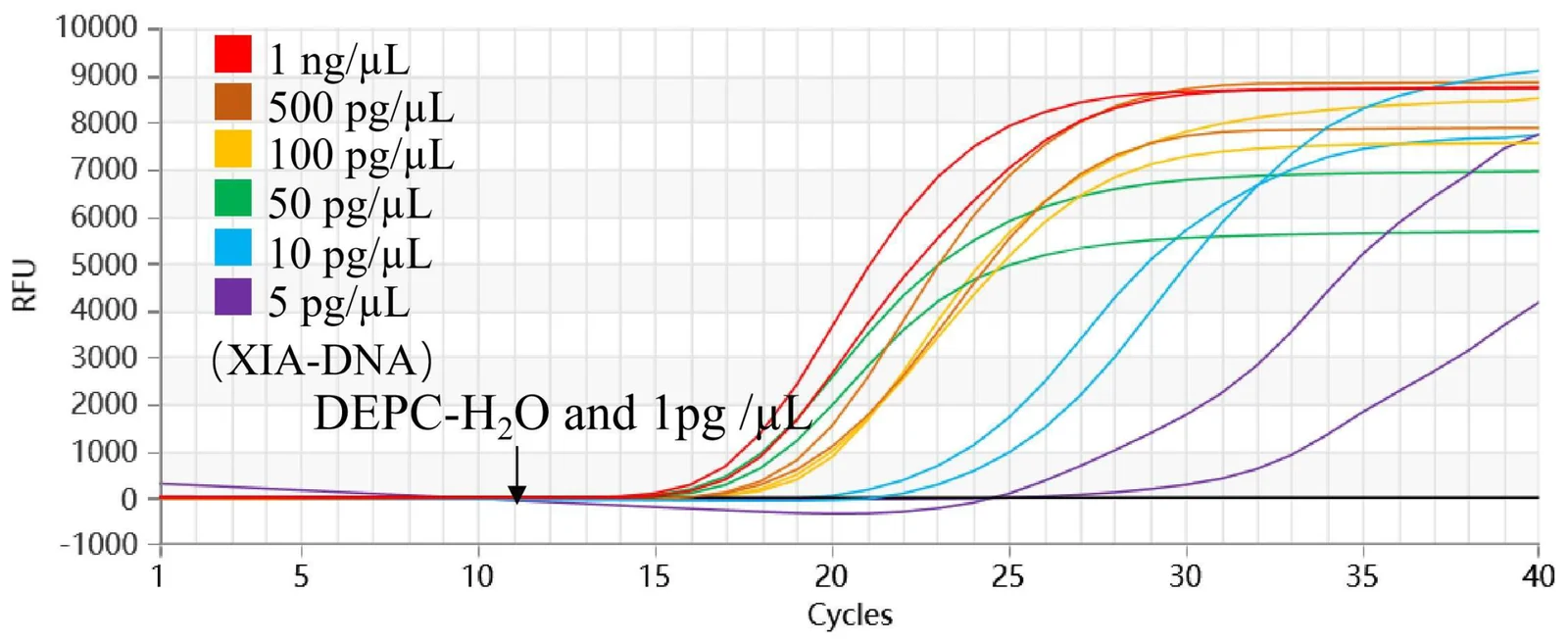
Analytical sensitivity of the reference LAMP assay for shrimp detection. Amplification curves for shrimp genomic DNA (gDNA) at concentrations of 1 ng/μL, 500 pg/μL, 100 pg/μL, 50 pg/μL, 10 pg/μL, 5 pg/μL, and 1 pg/μL. DEPC-H_2_O was used as the negative control. Xia: shrimp.

Figure 8 presents the analytical sensitivity results for the reference LAMP assay used to detect shrimp. Positive amplification signals were obtained for shrimp genomic DNA at concentrations of 1 ng/μL, 500 pg/μL, 100 pg/μL, 50 pg/μL, 10 pg/μL, and 5 pg/μL, whereas no amplification was observed at 1 pg/μL or in the DEPC-H_2_O negative control. Consistent amplification was observed among parallel reactions across the positive concentration range. Under the experimental conditions used, the minimum detectable concentration of shrimp genomic DNA using the reference LAMP assay was 5 pg/μL within a reaction time of 1 h.

## 4. Discussion

Among the aquatic products most widely consumed worldwide are shrimp, crab, and fish, which are valued for their delicate flavor and rich nutritional profile, including high-quality protein, vitamins, calcium, and iron. These foods also offer various health benefits; for example, shrimp contributes to healthy bone and tooth development, whereas omega-3 fatty acids may help lower cholesterol levels and reduce the risk of cardiovascular disease [7,27]. However, these foods are also highly allergenic and can trigger a wide range of allergic reactions following consumption. Clinical manifestations may include mild respiratory symptoms, such as asthma attacks and nasal mucosal inflammation; gastrointestinal symptoms, including abdominal cramps, nausea, and vomiting; and cutaneous reactions, such as pruritus and urticaria. They may also include severe and potentially life-threatening anaphylactic shock [28,29]. Food allergy incidence has continued to rise in recent years, and seafood allergy is particularly prevalent and severe, especially in Asian countries [30,31]. Moreover, seafood allergy represents an important challenge to the development of the seafood industry. Therefore, the establishment of efficient and accurate methods for seafood allergen detection is of great importance for the protection of consumer health [32].

A variety of analytical methods for seafood allergen detection have been developed in recent years. Suh et al. [33] reported a multiplex PCR method coupled with capillary electrophoresis that targeted the tropomyosin gene of oysters, mussels, abalone, and clams; the limit of detection (LOD) was 16 pg. For detection of fish DNA markers in food products, Cau et al. [34] developed a real-time PCR method targeting the fish *18S rRNA* gene. This method specifically detected 20 common fish species from the Mediterranean basin, with an LOD and limit of quantification (LOQ) of 0.5 pg/μL and 5 pg/μL, respectively. Subsequently, Cau et al. [35] developed a droplet digital PCR (ddPCR) assay targeting the same fish *18S rRNA* gene, achieving an LOD of 0.08 pg/μL and an LOQ of 0.31 pg/μL. Sheu et al. [36] developed a LAMP method for shrimp DNA detection. The LAMP primers showed high specificity, and amplification was completed within 30 min. The method did not cross-react with crab or lobster and detected shrimp at levels as low as 0.01%. For detection of the major shrimp allergen tropomyosin, Zhao et al. [37] developed sandwich ELISA methods based on polyclonal antibodies (PcAb) and monoclonal antibodies (mAb). The mAb-based assay showed the best performance, with an LOD and LOQ of 0.004 mg/kg and 0.006 mg/kg, respectively. Wang et al. [38] developed a quantum dot-based fluorescent lateral flow immunoassay (LFIA) targeting the major crustacean allergen tropomyosin, with an LOD of 0.5 μg/mL. For detection of molluscan tropomyosin allergens, Zhao et al. [39] developed a liquid chromatography–tandem mass spectrometry (LC-MS/MS) method. Using bioinformatics and liquid chromatography-high-resolution mass spectrometry (LC-HRMS), they identified two common peptides and group-specific markers, achieving an LOD of 1.5–3 mg/kg.

Although the methods reviewed above are applicable to seafood allergen detection, limitations inherent to many of them make it difficult to meet the increasing demand for rapid and convenient testing. Immunological methods such as ELISA and LFIA rely on the specificity of antigen–antibody interactions. Their performance is highly dependent on antibody quality, and cross-reactivity with non-target antigens may lead to false-positive results [9]. In addition, these assays may be affected by complex food matrices and intensive processing conditions. For example, high temperature and pressure can cause protein denaturation, potentially resulting in false-negative outcomes. Mass spectrometry (MS), although capable of providing high specificity and sensitivity even in complex matrices, requires expensive instrumentation, complex analytical procedures, and relatively high operating costs. Under processing conditions such as high temperature and pressure, DNA generally exhibits greater stability than proteins. However, conventional PCR requires post-amplification electrophoresis, making the procedure relatively labor-intensive. Although real-time PCR enables closed-tube detection, both methods depend on thermal cycling instruments, which limits their applicability for on-site testing. ddPCR, a relatively recent technique, offers high sensitivity and absolute quantification but also requires expensive instrumentation, restricting its use primarily to laboratory settings. LAMP is a well-established isothermal nucleic acid amplification technique. In the present study, LAMP demonstrated high sensitivity and stability, achieving an LOD of 5 pg/μL. Despite its incorporation into industry standards in many countries, LAMP requires 4–6 primers, is generally limited to uniplex detection, and remains susceptible to nonspecific amplification and false-positive results [40].

To address these limitations, a duplex Proofman-LMTIA method for detecting shrimp/crab and fish allergens was developed in this study. The method exhibited high analytical specificity, generating amplification signals for four shrimp species, ten fish species, and four crab species, thereby enabling broad detection of crustacean (shrimp/crab) and fish allergens. In addition, the duplex assay is simple to perform, requires only 30 min for reaction completion, and does not rely on thermal cycling, sophisticated instrumentation, or complex chemical analysis. To assess its specificity, the established duplex Proofman-LMTIA method was tested using squid, shellfish, and common meat species as negative controls, demonstrating high specificity for shrimp, crab, and fish. The assay detected shrimp and fish genomic DNA at 5 pg/μL and crab genomic DNA at 10 pg/μL; in meat mixtures, the limits of detection were 4% (*w*/*w*) for shrimp and 8% (*w*/*w*) for fish and crab. For further evaluation of the duplex assay in complex matrices, 12 commercial products containing fish, shrimp, or crab were analyzed. The results obtained for all 12 samples agreed fully with their labeled ingredient information, demonstrating the method’s accuracy and practical applicability in complex food matrices. The duplex assay achieved a minimum detectable concentration comparable to that of the reference LAMP assay while reducing the reaction time from 60 min to approximately 30 min and enabling simultaneous detection of two target groups.

Moreover, the duplex Proofman-LMTIA method established in this study is characterized by its simplicity, rapidity, and isothermal amplification format. The method has been successfully integrated with lyophilized microspheres and can be performed using a portable LAMP instrument, enabling on-site amplification. In the future, this approach could be further combined with microfluidic chip technology to achieve high-throughput and rapid detection.

For future commercial applications, further validation using a wider range of food matrices is needed to assess the stability and reproducibility of the assay. In addition, simplifying sample preparation, particularly DNA extraction, by developing more convenient and time-efficient extraction methods, improving reagent stability during storage and transportation, and further optimizing portable fluorescence detection systems would facilitate the practical application of this method.

## 5. Conclusions

In summary, this study successfully developed a duplex Proofman-LMTIA method for the simultaneous and rapid detection of crustacean (shrimp/crab) and fish allergens. Compared with existing standard LAMP methods, the proposed approach combines high sensitivity, high specificity, and robust stability with multiplex detection capability. The assay can be completed within 30 min and achieves minimum detectable genomic DNA concentrations of 5 pg/μL for shrimp and fish and 10 pg/μL for crab. In meat mixture systems, the LODs were 4% (*w*/*w*) for shrimp and 8% (*w*/*w*) for fish and crab, effectively reducing the risk of false-positive results. The isothermal amplification format, simple operation, and short detection time make the method particularly suitable for rapid on-site testing. Validation using commercial food samples demonstrated reliable and stable performance in complex food matrices. Overall, this work provides a valuable approach for addressing the growing public health challenges associated with food allergies.

## Figures and Tables

**Table 1:** GenBank database accession numbers of different species of shrimp, crab, and fish.

Types of Shrimp and Crabs	Accession	Fish Species	Accession
*P* *enaeus vannamei*	NC_091591.1	*Cyprinus carpio*	NC_001606
*Procambarus clarkii*	NC_091194.1	*Ctenopharyngodon idella*	NC_010288
*Cherax quadricarinatus*	NC_091352.1	*Siniperca chuatsi*	NC_015822
*Penaeus chinensis*	NC_061860.1	*Lateolabrax japonicus*	MW166212.1
*Homarus americanus*	NW_024745671	*Oreochromis niloticus*	NC_013750
*Penaeus indicus*	NW_026948844.1	*Trichiurus lepturus*	NC_062464.1
*Penaeus japonicus*	NW_025030369.1	*Carassius auratus*	EF483931.1
*Penaeus monodon*	NC_051420.1	*Channa argus*	NC_015191
*Panulirus ornatus*	NC_092257.1	*Salmo salar*	LC012541.1
*Oratosquilla oratoria*	NC_134800.1	*Pelteobagrus fulvidraco*	NC_015888.1
*Palaemon carinicauda*	NC_090740.1	*Gadus morhua*	NC_002081
*Macrobrachium nipponense*	NC_061110.1	*Pampus minor*	NC_032397.1

**Table 2:** Proofman-LMTIA primers and fluorescent probes.

Primers and Probes	Sequence (5′ to 3′)
XiaXie-F1	TTTCGTTAACCAGTTTTTGTGTTAATTGACCTGTGTTTGTTAAT
XiaXie-B1	AAAGGAGAAGTACAAGTTTTTAGTAGCCAGACAGTTCG
XiaXie-LF1	TCGTCTGCAAAGTAGA
XiaXie-LB1	CCGACGAGCTGGACCA
Yu-F2	ACAAACGAACCCTTAATTTTCCTCGATGTTGGATCAGGACA
Yu-B2	GTTCAACGATTAAATTTTACCTGGATTACTCCGGTCTGA
Yu-LF2	AGCGGCTGCACCATT
Yu-LB2	GTCCTACGTGATCTGA
XiaXie-Probe1	BHQ1-CCGACGAGCTGA-CY5
Yu-Probe2	BHQ2-GTCCTACGTGATT-FAM

Xia: shrimp (*Penaeus vannamei*); Xie: crab (*Portunus trituberculatus*); Yu: fish (*Piscis*).

**Table 3:** LAMP primers.

Primers	Sequence (5′ to 3′)
Xia-F3	TAGAAACCGACCTGGCTC
Xia-B3	TCATAAGATTAAGTTACTTTAGGGA
Xia-FIP	GCAGCAGTTATAAAGGAAGGTCT-CGGTCTGAACTCAAATCATGT
Xia-BIP	CCTTAATTCAACATCGAGGTCG-ACAGCGTAATCTTCTTTGAGAG

**Table 4:** Duplex Proofman-LMTIA reaction system (10 µL).

Composition	One Tube (μL)
LMTIA premix buffer (1×)	3
Bst Polymerase (100 U/µL)	0.1
XiaXie-F1 (100 μM)	0.16
XiaXie-B1 (100 μM)	0.16
XiaXie-LB1 (100 μM)	0.04
XiaXie-LF1 (100 μM)	0.04
XiaXie-probe1 (10 μM)	0.05
Yu-F2 (100 μM)	0.16
Yu-B2 (100 μM)	0.16
Yu-LF2 (100 μM)	0.04
Yu-LB2 (100 μM)	0.04
Yu-probe2 (10 μM)	0.5
Xia/Xie/Yu-DNA	2
DEPC-H_2_O	Add to 10 µL

Xia: shrimp (*Penaeus vannamei*); Xie: crab (*Portunus trituberculatus*); Yu: fish (*Piscis*).

**Table 5:** LAMP reaction system (25 µL).

Composition	One Tube (μL)
ThermoPol Buffer (10×)	2.5
Xia-F3 (5 μM)	1
Xia-B3 (5 μM)	1
Xia-FIP (40 μM)	1
Xia-BIP (40 μM)	1
dNTPs (10 mM)	5
Betaine (5 mol/L)	4
MgSO4 (100 mM)	0.5
Bst DNA polymerase (8 U/μL)	1
DNA template (10 ng/μL)	2
SYTO 9 (50 μM)	1
DEPC-H_2_O	Add to 25 µL

Xia: shrimp (*Penaeus vannamei*).

## Data Availability

The data that support the findings of this study are available up on request from the corresponding authors.

## Supplementary Materials

The following supporting information can be downloaded at https://www.mdpi.com/article/10.3390/foods15213842/s1, Figure S1. Schematic illustration of the ladder-shape melting temperature isothermal amplification (LMTIA) mechanism. (A) Primer melting-temperature profiles; (B, C) initial amplification stages; (D) exponential amplification stage. Figure S2. Temperature optimization of the singleplex Proofman-LMTIA assays. (A) Amplification curves for shrimp at 59–62 °C; (B) Amplification curves for fish at 58–61 °C. DEPC-H_2_O was used as the negative control. Figure S3. Market sample data figure.
