# Supplementary material for: A Duplex Proofman-LMTIA Assay for Rapid Detection of Shrimp, Crab, and Fish Allergens in Food Products
Source: Foods. 2026 Oct 27;15(21):3842. doi: 10.3390/foods15213842 (PMC13762627; doi:10.3390/foods15213842)
Supplement: Supplementary file 1 [file foods-15-03842-s001.zip › foods-4615566-supplementary.pdf]

Supplementary Material

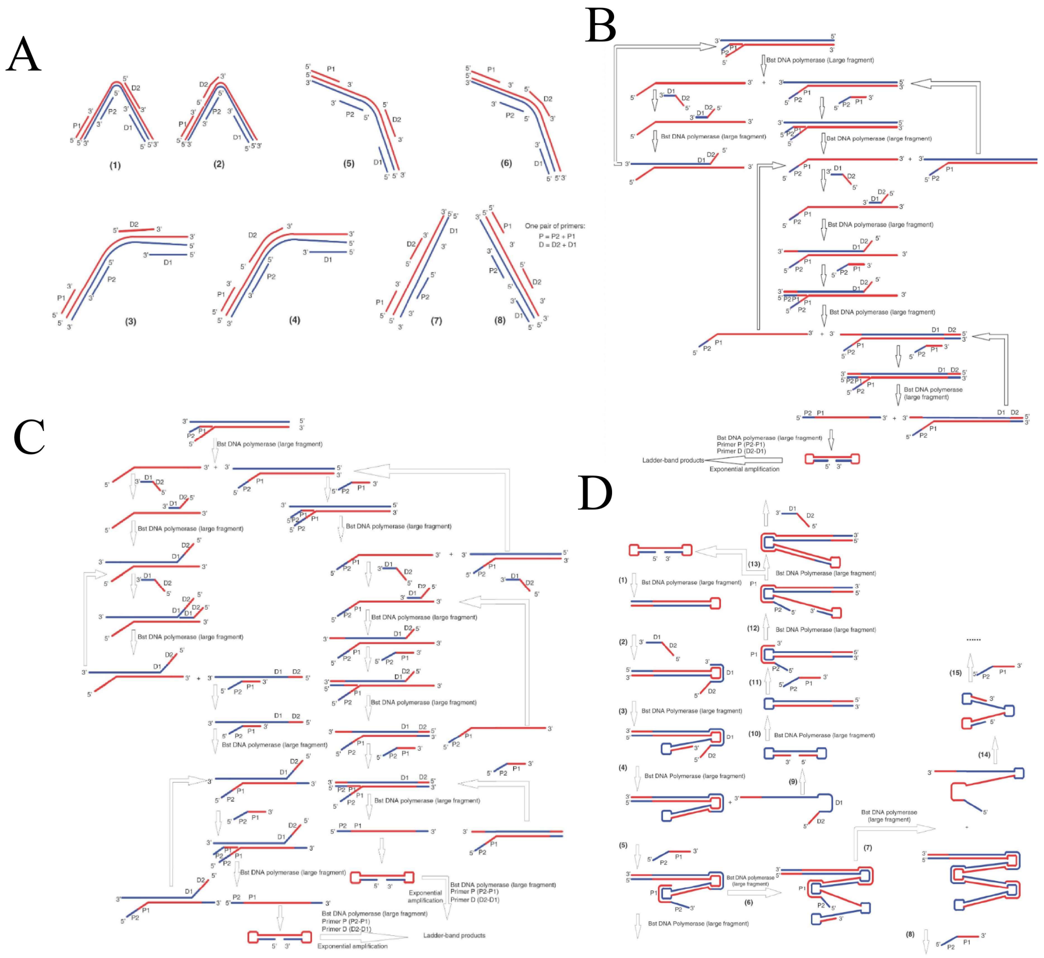

Figure S1. Schematic illustration of the ladder-shape melting temperature isothermal amplification (LMTIA) mechanism. (A) Primer melting-temperature profiles; (B, C) Initial amplification stages; (D) Exponential amplification stage.

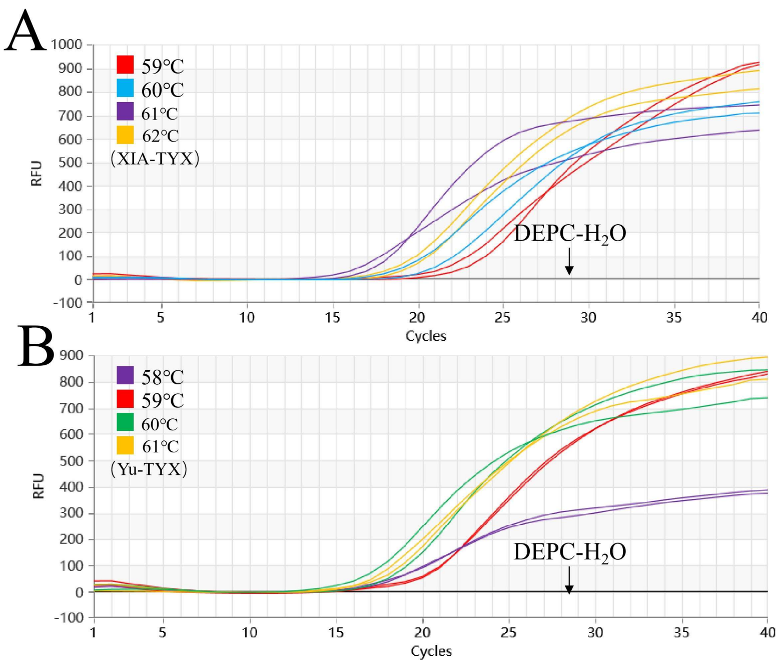

Figure S2. Temperature optimization of the singleplex Proofman-LMTIA assays. (A) Amplification curves for shrimp at 59–62 °C; (B) Amplification curves for fish at 58–61 °C. DEPC-H_2O was used as the negative control.

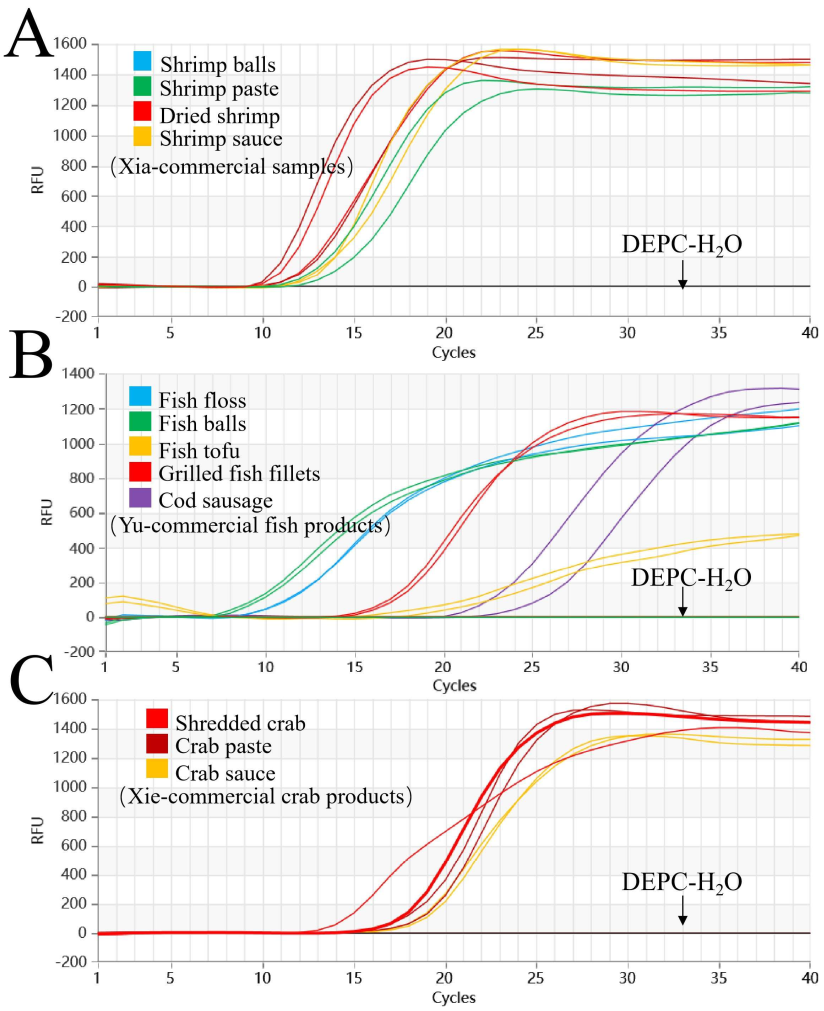

Figure S3. Market sample data figure
